# Persistent female genital mutilation despite its illegality: Narratives from women and men in northern Ghana

**DOI:** 10.1371/journal.pone.0214923

**Published:** 2019-04-22

**Authors:** Evelyn Sakeah, Cornelius Debpuur, Raymond Akawire Aborigo, Abraham Rexford Oduro, James Kotuah Sakeah, Cheryl A. Moyer

**Affiliations:** 1 Navrongo Health Research Centre, Navrongo, Ghana; 2 University of Calgary, Department of Medicine, Calgary, Canada; 3 University of Michigan, Ann Arbor, United States America; Kwame Nkrumah University of Science and Technology, GHANA

## Abstract

**Background:**

Globally, an estimated two million women have undergone Female Genital Mutilation (FGM), and approximately four percent of women who have been circumcised live in Ghana. In the Bawku Municipality and Pusiga District, sixty one percent of women have undergone the procedure. This study therefore aimed at identifying the factors that sustain the practice of FGM despite its illegality, in the Bawku Municipality and the Pusiga District.

**Method:**

This study used a descriptive qualitative design based on grounded theory. We used purposive sampling to identify and recruit community stakeholders, and then used the snowball sampling to identify, recruit, and interview circumcised women. We then used community stakeholders to identify two types of focus group participants: men and women of reproductive age and older men and women from the community. In-depth interviews and focus group discussions were conducted and qualitative analysis undertaken to develop a conceptual framework for understanding both the roots and the drivers of FGM.

**Results:**

Historical traditions and religious rites preserve FGM and ensure its continuity, and older women and peers are a source of support for the practice through the pressure they exert. The easy movement of women across borders (to where FGM is still practice) helps to perpetuate the practice, as does the belief that FGM will preserve virginity and reduce promiscuity. In addition, male dominance and lack of female autonomy ensures continuation of the practice.

**Conclusion:**

Female Genital Mutilation continues to persist despite its illegality because of social pressure on women/girls to conform to social norms, peer acceptance, fear of criticism and religious reasons. Implementing interventions targeting border towns, religious leaders and their followers, older men and women and younger men and women will help eradicate the practice.

## Background

Female Genital Mutilation (FGM) is deeply ingrained in many African societies. The practice occurs in 28 African countries with varying prevalence within and across countries [1]. Globally, an estimated two million women are circumcised [1] and four percent of those genitally mutilated live in Ghana. FGM is predominately practiced among the northern tribes of Ghana and the magnitude of the practice varies across regions and districts [2–4]. In the Bawku Municipality and Pusiga District, sixty one percent of women were reported to have undergone the procedure [5]. FGM has been in existence for many years, but it only generated international activism when it was widely discussed in the nineteen-eighties and early nineteen-nineties [6].

Historical antecedents and social research revealed that FGM was practiced by the Phoenicians, Hittites and ancient Egyptians. England and the United States used FGM to treat hysteria, lesbianism, masturbation and other female deviances in the 1940s and 1950s [7,8]. Higher occurrence of FGM in Northern Ghana has been attributed to the mixture of the people and culture of that part of the country with Burkina Faso, Togo and Mali, where the practice is more common. Some studies reported that traditions and social norms pressure girls to undergo circumcision: women who are not circumcised often experience ridicule from peers and rivals [2,9]. In some communities, the practice is tied to traditional religion [10] and in others, it is associated with Islam [11,12]. In some societies, circumcised girls receive mentorship to prepare them for marriage and meaningful lives. They are taught how to care for their families, homes, and uphold themselves in the community [2]. The belief is that circumcised girls can be trained on societal morals and values before they assume a larger role in the community as wives and mothers [9]. On the other hand, uncircumcised women risk being treated as men; their funeral and burial rites may be performed as if they are men because the presence of the clitoris makes a woman masculine [2]. The clitoris is also believed to induce aggressiveness in women and encourage promiscuous behavior [9]. This is very important in a culture where premarital and extramarital sex is morally unacceptable [13].

FGM was outlawed in Ghana in 1994, mandating punitive action against any perpetrator. The law stipulates that circumcisers and other perpetrators could be sentenced to not less than 3 years of imprisonment [14]. Although the law was drafted based on medical and social research that revealed dangers associated with the practice [15–18], it appears the law has not had much impact [19]. Understanding the reasons for the endurance of the practice in these settings is critical to designing intervention strategies to end the practice. This study therefore aimed at identifying the factors that sustain the practice of FGM despite its illegality, in the Bawku Municipality and the Pusiga District in the Upper East Region of Ghana.

## Study methods

### Methodology

The study employed grounded theory as its methodology. Grounded theory studies are mostly focused on social processes and actions and ask about what happens and how people interact [20]. This shows the influence of symbolic interactionism, a social psychological approach focused on the meaning of human actions [21]. Grounded theory studies begin with open questions, and researchers presume that they may know little about the meanings that drive the actions of their participants [20]. Therefore, we sought to learn from participants how and why FGM is still practice in the Bawku Municipality and Pusiga District despite its illegality. The generative research question which was how does the community view FGM (what meaning or value is attached to the practice), was opened and focused on social processes.

### Study setting and design

The study was conducted in the Bawku Municipality and the Pusiga District. Bawku is one of the municipalities that shares boundaries with Burkina Faso, the Republic of Togo, Bawku West District and the Garu-Tempane District to the north, east, west and the south respectively. The administrative capital is Bawku and the population of the municipality according to the 2010 Population and Housing Census stands at nearly 100,000 people, with slightly more women than men [22].

Pusiga shares boundaries with Burkina Faso to the North, the Republic of Togo to the East, the Bawku West to the West and Bawku Municipality to the South. The population of the district according to the 2010 Population and Housing Census stands at 57,677 [22].

This was a descriptive qualitative design based on grounded theory. We sought to explore the factors that contribute to the persistence of FGM in the Bawku Municipality and the Pusiga District and grounded theory as an exploratory model was deemed an appropriate methodology. We elicited rich descriptions of the practice of FGM from participants and gained insights into the factors that promote it in order to come out with variables for use in a survey that sought to measure the prevalence of the practice.

Grounded theory studies are characterized by theoretical sampling, but this requires some data to be collected and analysed [20]. Sampling must thus begin purposively, as in any qualitative study. We employed the purposive sample method to sample individuals and groups that were either associated with FGM or were identified to have information about the practice and invited them to participate in either an FGD or an IDI, and those typically included chiefs, community volunteers or women leaders. Community key informants facilitated the identification of men and women within the reproductive age (15–49 years old) for the initial interviews. Data from these FGDs suggested the next category of respondents to invite to participate in the study.

We conducted face-to-face IDIs and FGDs from the 18^th^ December 2017—17^th^ January 2018 in the local languages and transcribed them into English for analysis. We gathered data until saturation; where no new data emerged. Saturation was ensured through the constant comparative approach where data collected were constantly compared to previous data until the information became repetitive. This ensured that the data collected was exhaustive and trustworthy [20].

A total of 18 IDIs and 12 FGDs were conducted (see Table 1). The participants in the FGDs ranged from 8–10. All individual interviews were conducted in the homes of the respondents, whereas focus groups were conducted in places convenient to the participants and the interviews lasted 45 minute on average.

**Table 1:** Number of participants in the FGDs and IDIs.

Type of interview	Category of respondent	Age group	Bawku Municipal	Pusiga	No. Interviews
IDIs	Traditional leaders	40–70	1	1	2
	Elders	40–70	1	1	2
	Opinion leaders	35–65	1	1	2
	Women leaders	35–65	1	1	2
	Circumcised women	19–70	1	1	2
	Community volunteers	30–50	1	1	2
	Mothers	18–49	1	1	2
	Fathers	18–49	1	1	2
	Assembly members	30–50	1	1	2
**Total IDIs**					**18**
FGDs	Men of reproductive age	15–24		1	1
		25–34		1	1
		35–49	1		1
	Women of reproductive age	15–24	1		1
		25–34	1		1
		35–49		1	1
	Older women	50+	2	1	3
	Older men	50+	1	2	3
**Total FGDs**					**12**

#### Training and data collection

We recruited four university graduates with experience in qualitative data collection and trained them to conduct the IDIs and the FGDs. Training included both didactic sessions and mock interviews with real-time feedback to ensure neutrality in interviewing and the ability to probe effectively for additional information. The training lasted for two week after which the interviewers were sent to the field for the data collection. Interview guides were developed based on previous work [23] and were pilot tested in communities outside the study sites. We ensure that key terms were translated into the local languages to harmonize data collection between interviews. The interviews were recorded on an audiotape. Two experienced transcribers, who were not part of the interviewing team, transcribed, translated into English the data from the audiotapes before analysis of the transcripts. The interviewers reviewed the transcriptions for accuracy and completeness and corrected classification by questions to facilitate the work of the coding by theme.

We pre-tested the interview guides in the communities excluded from the study, but have similar characteristics, in order to improve the relevance and appropriateness of the questions. The pre-testing was a learning session for the research assistants to improve their interviewing skills, and we revised the guides appropriately after the pre-test.

#### Qualitative data analysis

The transcripts were reviewed for accuracy and completeness and corrected to facilitate the work of the coding by theme. The Principal Investigator (ES) and two other Co-Investigators (CAM and CD) sorted the transcripts by source and conducted multiple readings, writing memos in the margins of the text in the form of short phrases, ideas or concepts arising from the texts. We used these memos to iteratively develop coding categories. Discussions about the preliminary coding categories allowed us to identify themes that were recurrent across interviews as well as those that appeared to be atypical in response to each question. Transcripts were imported into NVIVO 11.0 for open, axial and selective coding by three separate coders (ES, CAM and CD). Coders met regularly to discuss the process of coding, revise the codebook as necessary and resolve any uncertainty in coding. Using grounded theory, thematic analysis was conducted, and themes were used to generate a conceptual model to understand both the roots of FGM and the subsequent facilitators.

#### Ethics approval and consent to participate

We obtained ethics approval for the study from the Institutional Review Boards of the Navrongo Health Research Centre and the University of Michigan. We also obtained written informed consent from all women and the Parents/guardians of those aged 15–17 years and assent from respondents aged 15–17 years prior to initiation of the interviews.

Community approval was obtained from chiefs and compound heads of the Bawku Municipality and Pusiga District prior to the study. Interview Participants were assured of anonymity and confidentiality before the interviews were conducted.

## Results

**Fig 1:**
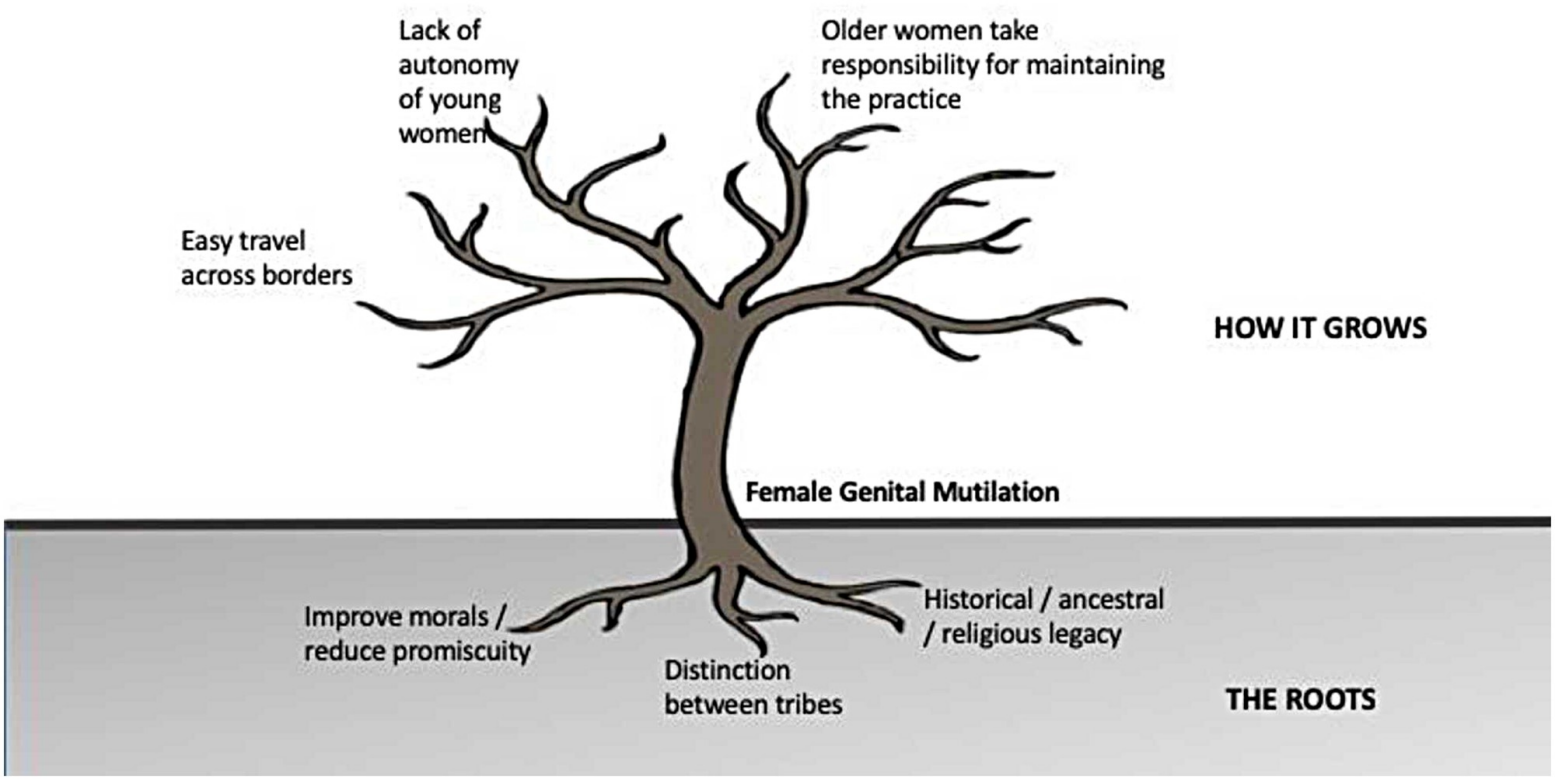
Conceptual model for FGM in rural northern Ghana.

Fig 1 illustrates the conceptual model that emerged from the data. The roots of FGM lie in historical / ancestral legacy, as well as a desire to draw a distinction between tribes and to ensure the virtuousness of the young women in the community. The drivers that facilitate FGM and perpetuate the practice include easy travel across borders, older women taking responsibility for maintaining the practice, and lack of autonomy of young women.

### The roots

#### Historical / ancestral / religious legacy

In IDIs with circumcised women and FGDs with women and men of reproductive age, it was generally believed that FGM started many years ago and could even be traced to the pre-historic era. While FGM may have been started by the ancestors of the modern-day residents of communities that still practice it, it was also clear that the practice was imported from neighboring countries such as Burkina Faso and Togo.

“…Female Genital Mutilation started a very long time ago, and it was practiced in Burkina Faso. It was used as a form of identification between people who were from Burkina Faso and those who were not.”

**(FGD with Men, 25–34 Years Old, Bawku Municipality)**

“…Female Genital Mutilation started from Adam and Hawa [Eve] over 2,000 years ago. It was started by our ancestors who then passed it on to our fathers and them to us. At the time, getting clothing was not easy so we and the women were wrapping leaves around our waists to serve as clothes.”

**(IDI with a Circumcised Woman, Pusiga District*)***

FGM is associated with tradition as it is understood as a rite of passage handed down by generations. Community members who uphold FGM strictly guard the practice to preserve its values and ensure continuity. FGD respondents believed that FGM is an important traditional practice that must be handed-down to generations yet unborn, compelling families to circumcise their daughters to satisfy this traditional requirement.

“… In my family for instance, it is a norm that any female child born must be circumcised, because it was handed over to us from our forefathers so we will also hand it over to the next generation.”

**(FGD with Men, 24–35 Years Old, Bawku Municipality)**

Traditional religion and Islam are observed as religions that require women to get circumcised. Thus, FGM is considered an element of these belief systems that must be preserved. According to the discussants, women are obliged to be circumcised because it is a religious requirement. According to community stakeholders:

“…We cannot stop FGM because we are Muslims and it was started by our great grandfathers therefore we cannot stop practicing it. If you are a female Muslim and you are not circumcised God will not accept your prayers because you are dirty, hence we have circumcised our females. If non-Muslims decide not to circumcise their females, we do not bother about that but for us Muslims we have to do it.”

**(FGD with Older Men, Bawku Municipality)**

“…Families from the traditional religion choose to circumcise their girls with the belief that it is a religious requirement.”

**(IDI with Community Health Management Committee member, Widana, Pusiga District)**

#### Drawing a distinction between tribes

In northern Ghana, some ethnic groups are identified with the practice. IDI and FGDs respondents confirmed that FGM is practiced among some tribes in the Bawku Municipality and Pusiga District and the practice distinguishes these tribes from non-practicing ones. Interview participants mostly linked the practice to particular tribes in the area, but also said other tribes adopted the practice overtime.

“…It is the tradition of the tribes so the girls cannot refuse to be circumcised. Can you see the tribal mark on my cheek? I am a Bissa and when I came from Kumasi I did not have any mark on my face, so my people insisted I have a tribal mark to show that I am a Bissa. I agreed that they could put one mark on my cheek but during the process they wanted to do more and I resisted which resulted in the mark being abnormal. When it comes to traditions, there is no compromise.”

**(IDI with Elder, Nwaare, Pusiga District)**

“…You know, this community is predominantly Busangas and it is we the Busangas who are much into the practice of FGM, therefore almost the whole community practices it.”

**(IDI with Traditional Leader, Bawku Municipality)**

“…It is the Busangas who encourage the practice of FGM, in the past we Kusasis did not know what circumcision was, when we started mingling with the Bisas that they influenced us to also circumcise.”

**(IDI with TBA, Kultimase, Pusiga District)**

#### Improve morals / reduce promiscuity

FGM is regarded as a practice that helps to preserve societal standards and ensures chastity among young women before marriage. Discussion of FGM among respondents revealed that adult women and men justify the practice as a mechanism that instills societal morals and values in young girls before they assume a larger role in the community as wives and mothers.

“… The reason why some women or girls continue to circumcise is that some of them grew up and heard that if you are not circumcised your level of sexual desire is high and you easily fall in love with men and that may make you promiscuous.”

**(FGD with Women, 15–24 Years Old, Bawku Municipality)**

### How FGM Grows

#### Easy travels across borders

Our results suggest that national borders are less important in defining zones for this practice than traditional tribal boundaries. For this reason, the practice continues to persist in border communities because women can easily travel across the borders to neighboring countries to circumcise their daughters. Respondents were of the view that the practice is sustained overtime because of collaborative activities among communities close to the borders.

“…I have heard of one FGM case related to my rival’s granddaughter about five months ago. The girl’s mother hails from Togo (Cinkasse) so she came and sent the girl there for the circumcision.”

**(FGD with Older Women, Widana, Pusiga District)**

“… I heard a story and I made my own investigation about a woman whose parents lived in Bador here, but married in Beituo [a town in Burkina Faso] and she will bring her children in Bador for the circumcision and later send them back to Beituo.”

**(IDI with a Father, Bawku Municipality)**

#### Older women and peers take responsibility for the practice

Mothers play an important role in the development of their girl-children. Their responsibilities include training, mentoring and preparing their girls for adulthood. One critical role of women is to ensure that their daughters are circumcised to prepare them for successful marriage lives and this gives them self-fulfillment and respect in society. These quotes summed up the views of discussants about the role women play in perpetuating the practice:

“…The pressure comes from the mothers of the children because men have no stake in it. Mothers think they will only be respected by their in-laws and other people in the community if they circumcised their daughters, so they will do whatever they can to circumcise them”

**(IDI with a Male Opinion Leader, Bawku Municipality)**

“…Yes, my husband’s family/tribe have about five (5) women who went through FGM and are currently and respectfully married to men of courtesy who show lots of respect to them as in-laws all the time. On the contrary, other girls of the family who are not mutilated genitally have gotten married to men who do not recognize their wives’ parents as their in-laws and will not even greet them when they meet or see them on the way. That is why mothers are keen on circumcising their girls.”

**(FGD with Older Women, Pusiga District)**

Similarly, IDI respondents identified older women as perpetrators of the practice, encouraging younger women to get circumcised because of perceived benefits associated with the practice.

“…It is the women, especially the older and illiterate women who still believe in some perceived benefits of female genital mutilation that supports the practice.”

**(IDI with Chief, Bawku Municipality)**

“…From my own observation, the older people, especially our grandmothers are still strongly in support of the practice.”

**(IDI with Community Health Management Committee Member, Bawku Municipality)**

In some communities, peers could exert powerful social pressure on a girl to undergo circumcision. This usually takes the form of insults, ridicule, isolation and name-calling, forcing their victims to succumb to their demands. In those communities, FGM is viewed as a requirement for peer social acceptance among friends, rivals and in the entire community, as the following quotes illustrate:

“… The point is that, some girls when they grow up and are not circumcised, their colleagues will be insulting them and this will make them go to circumcise themselves without even their parent’s knowledge.”

**(IDI with a Circumcised Woman, Bawku Municipality)**

“… Like the insult, if she is not circumcised and her colleagues are circumcised they will insult her, they will be insulting her in the form of a song. As they are insulting her, what does she have to do than to be circumcised? As her colleagues are circumcised and insulting her, she will say that as my colleagues are circumcised and insulting me, I will also get circumcised.”

**(FGD with Older Women, Pusiga District)**

A participant narrated how peer pressure led to the death of a girl who was circumcised

“…Even though it has been long, my brother’s daughter upon hearing that FGM was being done in Betu, bought her own razor blade and followed her peers in the night to the place. After undergoing the circumcision, they came back to the house but three (3) days later my brother’s daughter died. My niece met her untimely death because of peer pressure to get circumcised.”

**(IDI with an Elder, Bawku Municipality)**

#### Male dominance and female lack of autonomy

In parts of northern Ghana, some men prefer or marry women who have undergone the practice. Even in a situation where a man unknowingly marries an uncircumcised woman but later realizes it, he may divorce or send her back to the family to undergo the practice before she is accepted by her husband. Thus, in communities where marriage is very important to women, they have no alternative than to comply with the wishes of men. According to community stakeholders:

“…I heard of a story about a certain man, in that house all the women were circumcised so the man married a woman and when he was about to have sexual intercourse with her he realized that she was not circumcised, the man said that the woman would have to go back to her father’s house to be circumcised before he would accept her back as his wife.”

**(FGD with Men, 35–49 Years Old, Mandago, Pusiga District)**

## Discussion

This study was conducted to ascertain reasons why FGM still persists in the Bawku Municipality and Pusiga District. The resulting conceptual model illustrates that the roots of FGM lie in historical traditions and religious rites, the desire to draw a distinction between tribes, and the desire to ensure moral standards among the young women of the community. FGM is perpetuated by easy travel across borders, older women serving as a source of support for the practice, and lack of female autonomy.

FGM is embedded in religion, traditions and customs that perpetuate the practice and subject women to dehumanizing agony. This belief system justified the continuous existence of FGM because of the conviction that the practice has been handed down to community members by their ancestors [2,24–35] and fulfills religious obligations [30,31,36–42]. Yet, tradition and religion are dynamic and subject to change particularly when the very core values that they seek to preserve become threats to human existence. Some studies reported that FGM persists because it is regarded as a traditional practice that must be preserved [24,43]. Thus, ending the practice would require identification of the drivers of these traditions and a careful negotiation with their custodians.

The practice is seen as relevant because the clitoris is believed to induce aggressiveness in women and encourage promiscuous behavior [9,24], but FGM is believed to ensure and preserve girls’ or women’s virginity [28,30,37,44–48]. The expectation is that women are supposed to achieve societal morals and values [44] before they take larger roles in society as wives and mothers. In patriarchal society where women are expected to be bearers of children and good wives to their husbands, they have no other choice than to conform to societal norms and standards.

The practice continues to thrive in towns that share borders with Burkina Faso and Togo [49], the reason being that FGM was perceived to have been imported from these countries, where the practice was widespread. Since communities at the borders share the same historical background, traditions and customs, with their counterparts in Burkina Faso and Togo, it has become easy for community members from these settings to continue to elude established laws to cross the borders to circumcise their daughters because the tradition knows no boundaries. Halting the practice along border communities would therefore demand a cross-border approach. This is particularly crucial in the case of Ghana where laws against the practice are weaker in the neighboring countries where the practice is more profound. Other studies have reported high rise of cross-border FGM in West Africa [50–53].

Women also continue to play a central role in sustaining the practice in the two districts. They initiate the idea of circumcision with their daughters; orient them about perceived benefits of the practice and eventually get them circumcised. They do this because of the honour and respect they may receive after circumcising their daughters and also the belief that circumcised women or girls will get better suitors to marry [9,29,34,39,54,55]. However, it has been reported that women could still enhance their status through education and economic empowerment without FGM [54,56]. The continuing influence that women play in sustaining the practice could be refocused by creating status for themselves in a women’s society where power and influence is invested in programs that will enhance their livelihood and improve their participation in the decision making process on matters that affect their health [56]. Some analysts have suggested that women are the potential agents for social change [57] and so if they remain relatively open and supportive of programs, they could contribute significantly towards ending the practice [58]. In this view, women should be the targets for social action because they are a social resource that can foster the change needed to end FGM.

Again, derision by peers also sustains the practice in communities in the Bawku Municipality and Pusiga District. This is particularly disturbing when women crave for social acceptance among their peers, rivals and other people in their communities [55,59]. Positive deviants, who could act as agents of change, could help end the practice.

Patriarchy subjugates women and makes them second to men in societies where FGM continues to persist [44]. This system constrains women’s autonomy and gives men unlimited power to decide the fate of women on issues such as FGM [25,26,59–62]. Thus, male preference for circumcised women [24,54] compels them to undergo the practice without any negotiation or resistance. It is, therefore, believed that if men should show their preference for uncircumcised women publicly, the practice will come to a halt [53,63]. However, if women were empowered and engaged, that could reduce their overdependence on men for many things that include determining their circumcision status.

### Limitations

This study has a number of limitations. Using different local languages to collect the data could have distorted the presentation of the questions to the respondents. However, the standard training for fieldworkers and supervisors and the in-depth translation and back translation of the questions minimized the language bias. Also, the lead author as a female and a feminist could have introduced her biases in the discourse on FGM. However, she consciously sought to remain neutral and relied on the other authors’ contributions to ensure her biases were not unduly influencing the way questions were asked, the way data were collected, or the way it was analyzed and interpreted. Interviewers’ bias may have occurred since they came from the very communities, where the interviews were conducted. However, questions were perfectly neutral, giving no hint to the respondents of what they wanted to hear.

### Conclusion/Recommendations

Despite campaigns against FGM in the Bawku Municipality and the Pusiga District, FGM is still practiced. Tradition, ethnicity, religious beliefs and cross-border activities have contributed to the persistence of the practice in these settings. Older women and men have also contributed greatly to the perpetuation of the practice and the influence of peers has sustained the practice over the years. Therefore, implementing social interventions targeting community members particularly those close to the borders, the tribes that still practice it, men and women, adolescents and religious groups could help eradicate the practice.

## Abbreviations

FGM: Female Genital Mutilation
